# A Survey of Norwegian Nursing Students’ Responses to Student-Centered Small Group Learning in the Study of Human Anatomy and Physiology

**DOI:** 10.1177/23779608211045879

**Published:** 2021-10-04

**Authors:** Guanglin Cui, Jann-Briger Laugsand, Wei Zheng

**Affiliations:** 1 Faculty of Health Science & Nursing, 158927 Nord University, Campus Levanger, Levanger, Norway; 2 12636 The Second Affiliated Hospital of Zhengzhou University, Zhengzhou, China

**Keywords:** bioscience, student-centered learning strategies, small group learning

## Abstract

**Introduction:**

Small group learning (SGL) is a main learning strategy in the study of bioscience subjects in nursing schools.

**Objectives:**

We evaluated Norwegian nursing students’ responses to the student-centered SGL approach in the study of anatomy and physiology (A&P) and tried to determine what aspects educators should improve regarding the use of SGL in the study of biosciences.

**Methods:**

A descriptive questionnaire survey was conducted to evaluate Norwegian nursing students’ responses and experiences, for example, motivation, performance, satisfaction, and effectiveness of this new SGL strategy in the study of human A&P.

**Results:**

Nursing students showed a high motivation and varied experience, for example, different attendance rates, satisfaction, and effectiveness in response to the student-centered SGL strategy in the study of human A&P. In addition, some students reported a low completion rate of assigned work for each SGL session. Additional concerns were collected in the open-end survey section. Subsequent thematic analysis of these comments identified that SGL arrangement and teacher tutorials were the main themes that needed to be improved in future SGL practice.

**Conclusions:**

The information from this survey might provide new insights to educators to understand what and how we should improve the student-centered SGL work in future teaching practice.

## Introduction

In nursing schools, small group learning (SGL) with tutorials is one of the main approaches that is employed worldwide in the teaching/learning of bioscience subjects including anatomy & physiology (A&P) (Bartlett et al., 2020; Burgess et al., 2020; de Moura Villela et al., 2020; Grijpma et al., 2020; Meehan-Andrews, 2009). The size of an SGL group is usually between five students and eight students. The students can gather to study and discuss mentor-designed key questions in a bioscience subject (Wong, 2018). The role of teachers/mentors is to facilitate rather than direct students and to inspire rather than inform students (Mills & Alexander, 2013). However, previous studies have reported that nursing educators face many challenges in using SGLs to teach A&P. One of the main problems was how to establish a better way to activate students’ motivation and enhance the efficacy of SGL, to ensure a positive and pleasurable learning experience, and to facilitate the development of deeper learning outcomes (Barbosa et al., 2017; Johnston, 2010; Johnston et al., 2015; Knowles, 1985; Lujan & DiCarlo, 2006, 2015). Data are available from students regarding the traditional mentor-designed critical topics-based SGL in the study of A&P (Evensen et al., 2020). Students have complained that such an SGL approach consumed too much time for preparation and students in each SGL session only received one key question per organ system. Additionally, other important topics may be disregarded. Some students complained that they might waste SGL time on some mentor-deigned topics that they already well understood. Therefore, they expressed a strong desire to improve current SGL. As a result, we have introduced a student-centered SGL strategy to nursing students in the study of human A&P at our campus, in which students have a central and active role. This new student-centered SGL study strategy allows students to select the topics and determine how they should be discussed; teachers merely observe or may intervene and provide tutorials if necessary (Roberts, 2010). Since first-year nursing students had insufficient A&P knowledge, we combined this student-centered SGL form with Norwegian national learning outcome description (LOD) guidance issued by the National Agency for Quality Assurance in Education (NOKUT) in 2017. This national LOD listed all critical concepts and contents of A&P subjects in detail and has been utilized as a nationwide guideline for nursing students in the study of A&P in Norwegian nursing schools (Kvam et al., 2019). We postulated that this new student-centered and LOD-based SGL could result in improved motivation and learning activity, and become a supportive and effective learning approach in the study of human A&P.

## Objectives

We therefore designed and conducted this survey to evaluate nursing students’ responses and experiences with student-centered and LOD-based SGL learning strategies in the study of human A&P and to determine what aspects educators should improve in the use of SGL in the study of biosciences.

## Research Design/Methods

### Student-Centered SGL

First-year nursing students were divided into small groups of 5–6 students and studied A&P based on key issues listed in the Norwegian national LOD. Each SGL session lasted 2 h; one organ system was studied; 11 sessions were conducted. Students could select the topics and determine how they should be discussed; teachers merely observed or may have intervened and provided tutorials to students if necessary (Roberts, 2010).

### Setting and Participants

First-year nursing students at Campus Levanger, Nord University, and Central Norway participated in the data collection.

### Questionnaire Survey

A descriptive questionnaire survey was administered using our previously published forms and methods (Evensen et al., 2020). This survey contains 11 items, including students’ demographics (two items: age and gender), motivation (two items: motivation in the study of A&P and SGL) and performance (two items: attend rate and completion rate of assigned work) in student-centered SGL. The questions were adapted from Sturges D (2016) with some sectional modification (refer to Table 1). Items regarding teacher tutorials (two items), usefulness (one item), arrangement for current form (one item), and effectiveness (one item) of student-centered SGL are listed in Table 2. The statements of students’ responses to questions were mostly rated on a 3–5-point Likert scale from “strongly likely” to “strongly unlikely.” The final part of the questionnaire was an open section; the students could express any relevant comments that were not addressed in the questionnaire.

**Table 1.** Nursing Students’ Motivation in the Study of A&P (Q1) and Student-Centered Small Group Learning (Q2) ; the Rates of Attendance (Q3) and Completion of Assigned Task (Q4).

Questions	% (case/total case)	P
Q1: How likely are you to continue with your current A&P studying?		<.01
Very likely	56.4 (57/101)	
Somewhat likely	25.70 (26/101)	
Neither unlikely nor likely	10.9 (11/101)	
Somewhat unlikely	4.95 (5/101)	
Not at all likely	1.98 (2/101)	
Q2: How motivated are you to do well in student-centered SGL?		<.01
Very motivated	42.57 (43/101)	
Somewhat motivated	44.60 (45/101)	
Neither motivated nor unmotivated	10.89 (11/101)	
Somewhat unmotivated	0.99 (1/101)	
Unmotivated	0.99 (1/101)	
Q3: How often do you attend student-centered SGL (total 11 times)?		<.01
Every time	56.44 (57/101)	
Almost every time (1–2 missed)	38.6 (39/101)	
Most times (3–4 missed)	4.95 (5/101)	
Half times (5–6 missed)	0 (0/101)	
Q4: Could you complete all the questions in student-centered SGL every time?		<.01
Every time	12 (12/100)	
Almost every time (1–2 missed)	30 (30/100)	
Most times (3–4 missed)	29 (29/100)	
Sometimes (5–6 missed)	29 (29/100)	

Q = question.

**Table 2.** Teacher Tutorials (Q1 & 2) and Current Form of Student-Centered SGL (Q3 & 4).

Questions	% (case/total case)	P
Q1: I get helpfully feedback from teachers when I asked questions.		<.01
True all the time	22.77 (23/101)	
True most of the time	46.53 (47/101)	
True half of the time	16.83 (17/101)	
True little of the time	10.89 (11/101)	
True none of the time	2.97 (3/101)	
Q2: Tutorials helped me to understand the lecture materials during student-centered SGL session.		<.01
True all the time	11.88 (12/101)	
True most of the time	47.52 (48/101)	
True half of the time	25.74 (26/101)	
True little of the time	11.88 (12/101)	
Q3: Student-centered SGL allows me to interact and socialize with other students.		<.01
True all the time	48 (48/100)	
True most of the time	28 (28/100)	
True half of the time	18 (18/100)	
True little of the time	4 (4/100)	
True none of the time	2 (2/100)	
Q4: Current form of student-centered SGL (total 11 times).		<.01
Too much	23.76 (24/101)	
Generally acceptable	70.30 (71/101)	
Too little	5.97 (6/101)	
Q5: Is student-centered SGL useful for A&P study?		<.01
True all the time	10.89 (11/101)	
True most of the time	41.58 (42/101)	
True half of the time	28.71 (29/101)	
True little of the time	12.87 (13/101)	
True none of the time	5.94 (6/101)	

Abbreviations: SGL, small group learning; A&P, anatomy & physiology; Q, question.

### The Questionnaire and Data Collection

Data were collected in November 2018. A total of 102 first-year nursing students admitted in autumn 2018 from Campus Levanger, Nord University, and Central Norway were invited to participate in a 20-min brief class survey. Data were collected before the student-centered SGL was finished. To avoid influencing the student's responses since the lecturers were the researchers, the researchers were not present during the completion of the survey. Instead, two teachers who did not participate in this project were employed to distribute and collect the survey forms.

### Data Analysis and Presentation

Data were analyzed using Prism 5.0 and SPSS 27 software. Descriptive statistical treatment was utilized to compute the percentage for each item in the survey questionnaire. Cronbach's calculation was applied to validate the reliability analysis in eight items with a 5-point Likert scale in SPSS 27. The chi-squared (χ^2^) test was performed to analyze the differences among the answered scales for each item and *P* < .05 was considered statistically significant. An inductive thematic analysis was conducted to identify themes of submitted comments from students.

### Ethic Consideration

The study protocol was approved by the Faculty of Health Science & Nurse of Nord University prior to collecting data. A teacher informed the participants about the aim and procedure of this study. This study was voluntary and no incentives for participation were offered. Written informed consent was obtained from the participants.

## Results

### Students’ Response Rate and Demographics

We issued questionnaires to 102 students and 101 students completed the survey giving a response rate of 99.02% (101/102). To obtain an adequate sample size, the Raosoft Sample Size Calculator (Raosoft, 2004) was employed. The results showed that for a confidence level of 95% and a margin of error of 5%, a minimum sample size of 81 students was required. The actual sample size was 101; so, it was deemed to be statistically adequate. The demographics for the students in this student cohort are summarized in Table 3.

**Table 3.** Basic Information for Nursing Students Involving in the Questionnaire.

	Student-centered and LOD-based SGL group
Total issue cases	102
Response cases	101
Response rate (%)	99.02
Age range (year)^a^	18–34
Mean of age ± SE (year)	20.06 ± 0.42
Male/female ratio	13/88

^a^ In total seven students (six female and one male) submit their response without ages.

Abbreviation: LOD, learning outcome description; SGL, small group learning; SE, standard error.

### Different Motivations and Performances were Shown in Student-Centered SGL

Students’ motivation for studying A&P subjects and participating in student-centered SGL was investigated (see Table 1). The findings revealed that 56.43% (57/101) of students responded that they were “very likely” and 42.57% (43/101) responded “I am very motivated to do well.” However, 17.82% (11/101) of students were “neither unlikely nor likely,” 4.9% (5/101) were “somewhat unlikely” and 1.98% (2/101) were “not at all likely” to participate in student-centered and LOD-based SGL. These findings indicate that few students had low motivation in student-centered SGL.

Further data analysis revealed that the difference in motivation in nursing students produced a variation in SGC participation. This finding was reflected in a different rate of attendance in SGL. Although SGL was obligatory, only 56.44% (57/101) of students participated in every SGL session, 38.6% (39/101) participated in almost every SGL session, 4.95% (5/101) of students missed 3–4 SGL sessions and no one missed more than half of the SGL sessions (5–6 times). In addition, the completion rate of assigned work for each SGL session was low. For the assigned task in the national LOD, only 12% (8/100, one student did not answer this item) of the students completed every time, 30% (30/100, one student did not answer this item) of the students completed it almost every time; and 29% (29/100, one student did not answer this item) of students completed it most of the time (3–4 questions) and 29% (29/100, one student did not answer this item) sometimes (5–6 questions) completed it. This result might reflect the difficulty level of some of the items listed in the national LOD and the finding that many students needed additional time to complete the assigned task.

### Effectiveness of Teacher Tutorials and Satisfaction With Current Arrangements in Student-Centered SGLs

Regarding the effectiveness of teacher tutorials in the SGL study, most students were satisfied with the work of the teacher tutorials. The survey data showed that the students could obtain helpful feedback information from teachers when they asked questions (see Q1 in Table 2) and helped them to understand the lecture materials in the study of human A&P with SGL (refer to Q2 in Table 2). Survey data showed a variation in satisfaction rates for the current SGL strategy, which means that some students did not benefit from this new SGL format. In detail, 70.30% (71/101) of students thought the current student-centered SGL approach was generally acceptable. However, 23.76% (24/101) of students thought that there were too many sessions of current student-centered SGL. However, 5.97% (6/101) of students felt that the sessions were not sufficient and recommended increasing the SGL sessions in the future (refer to Q3 in Table 2).

Twenty-nine of the 101 students thought that student-centered SGL was successful half of the time, 19 students believed that it was successful only sometimes and 5 students felt that it was successful none of the time (refer to Q4 in Table 2).

To examine the reliability of the survey questions adapted from Sturges et al.(Sturges et al., 2016), the researchers performed a Cronbach's calculation on eight items with a 5-point Likert scale in SPSS 27 and obtained a value of α = 0.781. This result indicated that the reliability of the survey was generally acceptable.

### Additional Comments Submitted from Nursing Students

In the last section of open-ended questions, 24 students submitted their comments. To analyze the themes of the comments, an inductive thematic analysis of the responses was performed (refer to Table 4). Identified themes of submitted comments from students included the arrangement of SGL that is, time-consuming, frequency, inflexibility and ineffectiveness of SGL (n = 17). This result indicates that the arrangement of the SGL needs to be improved to meet the student needs individually.

**Table 4.** Thematic Analysis (Inductive) of Feedback Comments From Students.

Order	Feedback	Codes	Themes
1	I had more benefit from working with tasks than to work on LOD.	Task, LOD	Assigned task
2	I feel that the hippocampus fails me often and did not improve my study process. Therefore, I think it's hard for me.	Inflexibility and inconvenience, outcome	SGL arrangement and planning
3	Teacher cannot say “I do not know” when we asked questions.	Teacher tutorial	Involvement of teachers in SGL
4	SGL schedule does not work well, eliminating a lot of hours and noise for hours.	Schedule and time-consuming	SGL arrangement
5	The SGL starts too late.	Schedule	SGL arrangement
6	SGL Schedule does not work for me, and I feel that I did not benefit from the teacher tutorial.	Schedule and teacher tutorial	SGL arrangement, involvement of teachers in SGL
7	Unfortunately, I get a little in SGL hours. People were not motivated and just sit here to read themselves	Efficacy, motivation	SGL efficacy, motivation
8	Please describe key points more specifically.	Task key-points	Involvement of teachers in SGL
9	I don’t like the fact that the SGL is obligatory because exam was soon and I want to study on my own way. The SGL takes take too much time.	SGL inflexible, inconvenience and time-consuming	SGL arrangement
10	It is a pity that SGL is compulsory, as one learns in various ways and it is better to do tasks at home in their own way.	Compulsory and inconvenience	SGL arrangement
11	It seems silly that it is mandatory. We could do the same at home. Can we have multiple cohorts on the same day?	Mandatory and Inflexible	SGL arrangement
12	Teachers should post answers for assigned task after every SGL time.	Answer for task	Involvement of teachers in SGL
13	Sometimes the task is very complicated.	Task, complicated	Assigned task
14	It would be nice to have some presentations or posts for each other with different subjects so we could have some more active learning.	Presentation of tasks	SGL planning and arrangement
15	I like to know how we could eventually work on questions we got.	Work on task	SGL planning and tutorials
16	We might get more benefits if not mandatory, some topics I needed to work on alone.	Mandatory, studying form	SGL arrangement
17	I think that I would get more benefit from SGL if it were possible to work alone. Group work makes me unconcentrated.	Benefit and unconcentrated	SGL arrangement
18	Should we have had a different order on the SGL topics? It is better to arrange the hardest/biggest topics near the exam time.	Task order	Task schedule
19	Tasks in SGL are too easy, and we finished it in 1 h. Can we get more tasks or other activities?	Task easy	Assigned task
20	We have consumed a lot of time in SGL/LOD, so I would like to make the time less than current level.	Time-consuming	SGL arrangement
21	SGL costs too much and no time for self-study.	Time-consuming	SGL arrangement
22	Light teacher tutorial, too much obligatory hours, less time for self-preparation for exams.	Time-consuming and involvement of teacher	SGL arrangement
23	Too much obligatory hours and not time for individual study, often supervisors without sufficient knowledge for tutorial during the SGLs.	Inflexibility and inconvenience and time-consuming, involvement of teacher	SGL arrangement and involvement of teachers in SGL
24	Too much time for SGL and little time for the preparation of final exams.	Time-consuming	SGL planning and arrangement

Abbreviations: SGL, small group learning; LOD, learning outcome description.

The assigned tasks for each SGL session comprise another theme that was often reported. In this section, four students submitted their comments that stated that the assigned tasks would help students more than LOD. Reordering the time/sequence of tasks according to the difficulty level might also help students to prepare for an exam. The involvement of teachers (teacher tutorials) in the SGL was an important factor in influencing the development of deep learning outcomes. However, the theme analysis revealed that two students complained that the quality of some teacher tutorials in the SGL study of A&P was low and that sometimes the students could not obtain the correct answers from teachers when they asked questions.

## Discussion

Evaluating learning/teaching approaches employed in nursing education is important for the improvement of learning outcomes (Thrower et al., 2020). Although a benefit of SGL has been clearly demonstrated in pedagogical studies of bioscience subjects (Burgess et al., 2020; Cunningham & Zlotos, 2019; de Moura Villela et al., 2020; Grijpma et al., 2020; Stewart & Cunningham, 2020), concerns many publications on SGL put too much emphasis on the role of the teacher (mentor) and did not put enough emphasis on the response of the students (Edmunds & Brown, 2010; Mills & Alexander, 2013; Parmelee, 2011; Roberts, 2010). Therefore, we surveyed nursing students’ motivation, performance, satisfaction, and effectiveness regarding student-centered SGL in nursing students during the study of A&P. Our survey data revealed that student-centered SGL is generally a good learning approach and can help students enhance student learning by fostering critical thinking on vital topics and concepts. However, several aspects need to be improved to obtain an enhanced understanding and deep comprehensive realization of bioscience.

First, to validate the reliability of the survey adapted from Sturges et al. (2016), the researchers performed a Cronbach's calculation on eight items with a 5-point Likert scale in SPSS 27. The calculation revealed that the value of α was 0.781. This result indicates that the reliability of survey questions is generally acceptable.

One of the important features of SGL is that all students actively participate in the group discussion and contemplate the assigned work in every SGL session (Edmunds & Brown, 2010; Jackson et al., 2014; Mills & Alexander, 2013; Parmelee, 2011). However, this aim would be difficult to achieve if the students are unmotivated (Maurer et al., 2013; Sturges et al., 2016). These survey data revealed different motivations among nursing students in student-centered SGL. Almost 18% of students were unmotivated, indicating that some students did not realize the importance of A&P subjects for their nursing program and future career development. Current survey data of motivation were similar to the findings of a study conducted by Evensen et al. (2020), which indicated a high motivation among Norwegian nursing students in the study of A&P (Evensen et al., 2020). Further data revealed that the rate of attendance in student-centered SGL sessions varied. Although the SGL was obligatory, only 56.44% (57/101) of students attended every session of SGL. The attended rate of Norwegian nursing students was slightly higher than the reported rate of African nursing students in the study of A&P (Mhlongo & Masango, 2020). These finding might be attributed to the different composition of students; African nursing students comprise a nonhomogeneous group, and many students live in rural areas far from their school (Mhlongo & Masango, 2020). In addition, the completion rate of assigned work in each SGL session was low, and only 12% (8/100) of students completed the work every time. These findings might reflect the difficulty level of A&P content for the majority of first-year nursing students.

In student-centered SGL, the students could select the topics and determine how they should be discussed. Traditionally, mentor-designed tasks are distributed to students during SGL sessions (Jackson et al., 2014). However, an A&P course includes a mixture of anatomy and physiology, containing many concepts and subjects. The mentor-designed topics only cover select topics and students might disregard other information during an SGL session. The national LOD designed by NOKUT covers in detail all the key content that students need to learn and control. The national LOD provides students with a wide range of information and tasks for the SGL study of A&P and helps students to comprehensively understand and control the necessary and key A&P information. By studying the national LOD for SGL sessions, students could systematically understand, focus, and discuss the key content in each organ system. Therefore, the researchers applied the LOD as a task in the current student-centered SGL study of A&P. Usually, one system was explored during each SGL session. Compared to the almost 100% satisfaction rate of Saudi Arabian nursing students in the SGL strategy (Megahed & Mohammad, 2014), the satisfaction rate (true all the time +  true most of the time = 52.47%) of Norwegian nursing students was lower. Such differences might be attributed to the different sample sizes, cultures and bioscience subjects.

Previous studies have revealed that physiological sections, particularly sections on the regulatory mechanisms of physiological functions, were more difficult for students than the anatomical sections (Evensen et al., 2020). To help students understand the key materials and learn, teachers therefore need to intervene and provide tutorials to students during the relevant SGL sessions (Exley et al., 2019; McKimm & Morris, 2009). The survey data showed that approximately 70% (70/101) of students were satisfied and 30.7% (31/101) of students were unsatisfied with current teacher tutorials during the SGL sessions. A thematic analysis of submitted comments from students revealed that one possible reason for this analysis might be that “the teacher could not give an appropriate answer to the students,” which reflects an inadequate preparation of teachers for tutorials.

In the last open-end survey section, we received feedback comments from 24 students. By thematic analysis, the researchers identified that one of the most frequent themes is the arrangement of the SGL, that is, time consumption, inflexibility, and ineffectiveness and efficacy of SGL. This finding reminds educators that an improved and more flexible SGL format is needed. Assigned tasks with SGL have a critical role in SGL learning and comprise another frequent theme in the submitted comments. How to order the sequence and difficulty level of tasks should be improved in future SGL studies. Another theme is the involvement of teachers in the student-centered SGL. Some students complained that some teachers could not give appropriate help when students needed help on assigned topics. This outcome reflects that some teachers did not receive appropriate tutor training before they became involved in SGL; therefore, sufficient training on how to be an effective SGL teacher and familiarity with how to guide students in the student-centered SGL is important. The analysis of these comments from students will help educators improve SGL and make it more personalized to fulfill the individual needs of nursing students in student-centered SGL in the future.

### Limitations

In this study, several limitations must be addressed. The participants in this survey were recruited from a single campus. According to the national registration data of the entry grade point average (GPA) in Norwegian universities, the entry GPA of nursing students was higher on this campus than on other campuses or at most Norwegian nursing schools. Whether current findings can be extrapolated to the whole Norwegian nursing student population remains unknown. Therefore, validation of data obtained from other campuses or Norwegian nursing schools is urgently needed. Furthermore, the researchers did not consider the age difference in students between the groups and allowed students to freely select SGL group members that might make them feel more comfortable. However, such an SGL group division method might influence the study effectiveness, as it is known that the age of students is one major factor influencing learning outcomes (Cho et al., 2010). Therefore, it is important to compare the effectiveness of SGLs for students of different ages in the future. Educators should consider how to identify nursing students with surface learning early to provide extra tutorials to these students during the relevant SGL sessions.

### Implications for Education Practice

The information obtained from this study provides new insights that can help educators understand what nursing students have experienced in the use of SGL as a learning strategy in the study of A&P and how SGL work can be improved for future bioscience teaching practice. For example, inductive thematic analysis of the comments from students revealed that the planning and arrangement of SGLs was time-consuming, inflexible, and ineffective for some students. Table 3 also demonstrates that only 56.44% (57/101) of students attended every SGL session. Can students attend the group SGL session in a flexible model? The experience and effectiveness of online teaching approaches using Zoom and Microsoft Teams during the coronavirus disease 2019 (COVID-19) pandemic period have given us good examples and might provide an option for educators and students to utilize SGL in a flexible and convenient way. The new SGL structure does not require the students to sit in a classroom on campus. The students can find a time that is suitable for them to complete the SGL with assigned tasks via Zoom or Microsoft Teams. The teachers observe and guide the SGL and provide tutorials to students with assigned topics via an internet video camera if necessary. Furthermore, the thematic analysis of comments from students revealed that a few teachers involved in the guidance of SGL lacked sufficient training and A&P knowledge. They could not give students appropriate help when the students needed it during an SGL session. This finding indicates that sufficient training should be given to teachers before they can participate in SGL guidance. The difficulty of assigned work for each SGL session needs to be modified. Each SGL session lasted only 2 h; therefore, it would be difficult to complete the session if the difficulty level of assigned work was too high or the amount of work was excessive. Educators need to divide some systems with difficult topics into 1.5–2 SGL sessions in the future.

## Conclusion

The results of this study suggested that Norwegian nursing students presented varied experiences in student-centered SGL for several aspects, for example, motivation, performance, satisfaction, and understanding and realization of key content in the study of human A&P. Such feedback information is important for educators to improve student-centered SGL work and to further personalize it to future nursing education.
